# Long non-coding RNA H19 enhances the pro-apoptotic activity of ITF2357 (a histone deacetylase inhibitor) in colorectal cancer cells

**DOI:** 10.3389/fphar.2023.1275833

**Published:** 2023-09-28

**Authors:** Chiara Zichittella, Marco Loria, Adriana Celesia, Diana Di Liberto, Chiara Corrado, Riccardo Alessandro, Sonia Emanuele, Alice Conigliaro

**Affiliations:** ^1^ Department of Biomedicine, Neurosciences and Advanced Diagnostics (Bi.N.D.), Section of Biology and Genetics, University of Palermo, Palermo, Italy; ^2^ Department of Biomedicine, Neurosciences and Advanced Diagnostics (Bi.N.D.), Biochemistry Building, University of Palermo, Palermo, Italy; ^3^ Institute for Biomedical Research and Innovation (IRIB), National Research Council (CNR), Palermo, Italy

**Keywords:** lncH19, colorectal cancer, histone deacetylase inhibitor, apoptosis, drug resistance

## Abstract

**Introduction:** Long non-coding RNA H19 (lncH19) is highly expressed in colorectal cancer (CRC) and plays critical roles in tumor development, proliferation, metastasis, and drug resistance. Indeed, the expression of lncH19 usually affects the outcomes of chemo-, endocrine, and targeted therapies. ITF2357 (givinostat) is a histone deacetylase inhibitor (HDACi) that revealed a significant anti-tumor action by inducing apoptosis in different tumor models, including leukemia, melanoma, and glioblastoma. However, no data are present in the literature regarding the use of this compound for CRC treatment. Here, we investigate the role of lncH19 in ITF2357-induced apoptosis in CRC cells.

**Methods:** The HCT-116 CRC cell line was stably silenced for H19 to investigate the role of this lncRNA in ITF2357-induced cell death. Cell viability assays and flow cytometric analyses were performed to assess the anti-proliferative and pro-apoptotic effects of ITF2357 in CRC cell lines that are silenced or not for lncH19. RT-PCR and Western blot were used to study the effects of ITF2357 on autophagy and apoptosis markers. Finally, bioinformatics analyses were used to identify miRNAs targeting pro-apoptotic factors that can be sponged by lncH19.

**Results:** ITF2357 increased the expression levels of H19 and reduced HCT-116 cell viability, inducing apoptosis, as demonstrated by the increase in annexin-V positivity, caspase 3 cleavage, and poly (ADP-ribose) polymerase (PARP-1) degradation. Interestingly, the apoptotic effect of ITF2357 was much less evident in lncH19-silenced cells. We showed that lncH19 plays a functional role in the pro-apoptotic activity of the drug by stabilizing TP53 and its transcriptional targets, NOXA and PUMA. ITF2357 also induced autophagy in CRC cells, which was interpreted as a pro-survival response not correlated with lncH19 expression. Furthermore, ITF2357 induced apoptosis in 5-fluorouracil-resistant HCT-116 cells that express high levels of lncH19.

**Conclusion:** This study shows that lncH19 expression contributes to ITF2357-induced apoptosis by stabilizing TP53. Overall, we suggest that lncH19 expression may be exploited to favor HDACi-induced cell death and overcome 5-fluorouracil chemoresistance.

## Introduction

Accumulating evidence indicates that long non-coding RNAs (lncRNAs) profoundly influence cancer development through intricate networks based on their interplay with DNA, RNAs, and proteins. LncRNA-H19 (lncH19) is one of the first lncRNAs identified and exerts multiple functions in various diseases, including cancers (Bao et al., 2018; Bitarafan et al., 2019; He et al., 2020; Yang et al., 2021). LncH19 is canonically considered to exert an oncogenic function since it is upregulated in many forms of tumors and is associated with tumor transformation, progression, and malignancy (Shima et al., 2018; Corrado et al., 2019; Mahmoudian-Sani et al., 2019; Zhou et al., 2019). LncH19 may also act through the production of intragenic microRNAs, miR-675-5p and miR-675-3p, which also display a pro-tumor activity (Lo Dico et al., 2016; Muller et al., 2019). LncH19 and its intragenic miRNAs are upregulated in colon tumors and correlate with poor prognosis in patients (Costa et al., 2017; Feng et al., 2017; Zhang et al., 2017; Dai et al., 2019; Yang et al., 2020; O'Brien et al., 2022).

In colorectal cancer, lncH19 overexpression affects cell proliferation (Yang et al., 2017; Saieva et al., 2020) and cell motility (Ding et al., 2018; Yang et al., 2018), and more recently, scientific evidence correlates the expression levels of lncH19 with the reduced sensitivity to 5-FU, suggesting that lncH19 may function as a marker for prediction of the chemotherapeutic response to this drug (Wang et al., 2018; Zhang et al., 2022).

Wang and collaborators demonstrated that lncH19, functioning as a competitive endogenous RNA, mediates 5-FU resistance in CRC via SIRT1-mediated autophagy (Wang et al., 2018).

We have recently demonstrated that lncH19-derived miR-675-5p enforces hypoxia-induced chemoresistance to 5-FU by targeting pro-caspase-3 and inhibiting the pro-apoptotic effects of 5-FU (Zichittella et al., 2022).

Numerous studies propose the therapeutic use of histone deacetylase inhibitors (HDACis) for the treatment of several diseases, including metabolic, inflammatory, autoimmune, and neurodegenerative diseases, and not least for the treatment of cancer (Eckschlager et al., 2017; Vagapova et al., 2021; Squarzoni et al., 2022).

HDACis are well-known epigenetic drugs with widely recognized anti-tumor activity (Zhao et al., 2020). HDACis target the aberrant activity of histone deacetylases (HDACs), which are often overexpressed in tumor cells, restoring or increasing histone acetylation, thereby promoting transcriptional activation of tumor suppressor and pro-apoptotic genes (Singh et al., 2018; Patra et al., 2019; Ramaiah et al., 2021). Therefore, inhibition of HDACs represents a valid basis for new anti-tumor therapies (Dasko et al., 2022).

To date, the Food and Drug Administration has approved some HDACis such as vorinostat (SAHA), belinostat (PXD-101), panobinostat (LBH-589), and romidepsin (FK-228) for the treatment of cancer (Squarzoni et al., 2022). Clinical and pre-clinical studies have also shown that these compounds can be used as adjuvants to traditional chemotherapeutics in different types of cancer (Suraweera et al., 2018; Psilopatis et al., 2021; Pramanik et al., 2022). More recently, it has been shown that epigenetic targeting of colon cancer based on combined HDACis with DNA methyltransferase (DNMT) inhibitors has revealed clinical relevance (Tang et al., 2023).

ITF2357 (givinostat) is a potent HDAC inhibitor belonging to the hydroxamic acid class. This compound is currently used in the therapy for the treatment of Duchenne muscular dystrophy, and in clinical trials for Becker muscular dystrophy and juvenile idiopathic arthritis (Vojinovic and Damjanov, 2011; Vojinovic et al., 2011; Spreafico et al., 2021; Comi et al., 2023; Sandona et al., 2023).

The compound has also revealed a significant anti-tumor action by inducing apoptosis in different tumor models, including leukemia, melanoma, and glioblastoma cells (Li et al., 2016; Celesia et al., 2022; Taiarol et al., 2022).

In addition, it has been widely demonstrated that ITF2357 can also act as an adjunct to conventional chemotherapy, increasing sensitivity to demethylating or chemotherapeutic agents such as pemetrexed in lung cancer, doxorubicin in sarcoma cells, and temozolomide in glioma stem cells (Di Martile et al., 2018; Cui et al., 2023; Nakagawa-Saito et al., 2023).

ITF2357 has recently been reported to exert a targeting effect on oncogenic BRAF in melanoma cells (Celesia et al., 2022) and affect oncogenic BRAF and p53 interplay, thus representing a promising candidate for melanoma-targeted therapy (Celesia et al., 2023).

To date, the only data present in the literature on the effects of ITF2357 in colon cancer are described in a manuscript that discusses the use of the compound for the prevention of colitis-associated cancer in mice (Glauben et al., 2008). Here, we describe the pro-apoptotic effect of ITF2357 in CRC cells and show that lncH19 plays a functional role in apoptosis execution by stabilizing TP53, probably by exerting its action as a miRNA sponge. Moreover, the paper provides evidence that lncH19-expressing CRC cells, resistant to 5-FU treatment, nicely respond to ITF2357, thus supporting a possible therapeutic application of this compound to overcome colon drug resistance.

## Materials and methods

### Cell culture

HCT-116 cells (ATCC–LGC Standards S.r.L., Italy) were cultured in McCoy’s 5A medium (Euroclone, United Kingdom) supplemented with 10% fetal bovine serum, 1% penicillin/streptomycin (10,000 U/mL penicillin and 10 mg/mL streptomycin), and 200 mM L glutamine (all sourced from Euroclone, United Kingdom).

5-Fluorouracil (5-FU)-resistant HCT-116 cells (HCT-116-5-FU-R) were cultured in DMEM (Euroclone, United Kingdom) supplemented with 10% fetal bovine serum, 1% penicillin/streptomycin (10,000 U/mL penicillin and 10 mg/mL streptomycin), and 200 mM L glutamine (all sourced from Euroclone, United Kingdom), and additionally, the culture medium contained 5-fluorouracil (5-FU, cat. n°F6627, Sigma-Aldrich, St. Louis, MO, United States) at concentrations up to 70 µM.

Cells were maintained in a humidified atmosphere containing 5% CO_2_ at 37°C and used at early passages for all experiments. The culture medium was changed every 2–3 days, and cells were split at 70%–80% confluence.

### Infection with lentiviral vectors to stably silence lncH19

HCT-116 cells were stably silenced for lncH19 by lentiviral infection with H19 human shRNA lentiviral particles (Cat. n° TL318197V, OriGene Technologies, Inc., Rockville, MD, United States), while relative control cells were infected with control shRNA lentiviral particles (Cat. n° TR30021V, OriGene Technologies, Inc., Rockville, MD, United States). Subsequently, infected cells were selected by cell sorting (BD FACSAria™ III Sorter, ATeN Center) and maintained in culture under selective pressure with 1 mg/mL of puromycin (Gibco™ puromycin dihydrochloride, cat. n°A1113802, Thermo Fisher^®^ Scientific, United States). Silencing efficiency was regularly tested by qRT-PCR and fluorescence microscopy.

### Selection of HCT-116-5-FU-resistant cells

The 5-FU-resistant HCT-116 cell line (HCT-116-5-FU-R) was established after sequential treatments with 5-FU during an 8-month period starting from 1 μM to 70 µM concentrations. Control parental cells were split in parallel. Viable cells treated with 70 µM 5-FU were considered stably resistant when the morphology resembled that of parental HCT-116.

### Chemicals and reagents

ITF2357 (givinostat) was synthesized and supplied by the pharmaceutical company Italfarmaco S.p.A. (Cinisello Balsamo, MI, Italy). For *in vitro* experiments, ITF2357 was dissolved in DMSO (20 mM stock solution) and stored at −20°C. Before use, the stock solution was thawed and diluted in McCoy's 5A or DMEM culture media, not exceeding 0.01% (v/v) DMSO, to realize the proper final concentrations.

The autophagy inhibitor bafilomycin A1 (Cat. n° B1793-2UG, Sigma-Aldrich, United States) was solubilized in DMSO, according to the data sheet instructions and used for the experiments at 20 nM and 50 nM final concentrations.

### MTT [3-(4,5-dimethylthiazol-2-yl)-2,5 diphenyl tetrazolium bromide] assay

Cell viability was determined by MTT assay, following the manufacturer’s instructions (Cat. n° M6494, Thermo Fisher^®^, United States), and the absorbance was measured using a biophotometer at 540 nm (BioTek Elisa ELx800 Absorbance Microplate Reader, BioTek Instruments, United States).

HCT-116 cells (wild type, silenced for lncH19, or 5-FU-resistant) were seeded in at least three technical replicates at 5 × 10^4^ cells/cm^2^; then, 24 h post-seeding, cells were treated with different concentrations of ITF2357 (0.25–0.5–1–2 µM or 4 µM) and maintained in a humidified atmosphere of 5% CO_2_ at 37°C. The MTT assay was performed at different time points, as indicated in the results.

For the experiments with the autophagy inhibitor bafilomycin A1, HCT-116 cells were pretreated for 1 h with bafilomycin A1 (20 nM and 50 nM concentrations), and then, ITF2357 was added at different concentrations (0.25–0.5 µM or 1 µM) for 48 h.

### Colony formation assay

LncH19-silenced HCT-116 cells and control cells were seeded at 40 cells/cm^2^ in six-well plates. After 48 h, cells were treated with different concentrations of ITF2357 (0.05–0.1–0.25 µM and 0.5 µM) and maintained in culture for 8 days to allow clone formation. Clones were then washed once with phosphate buffer solution (PBS), fixed, and stained with methylene blue 1% in PBS/ethanol 50% for 1 min at room temperature. Following air-drying, clones were observed under a light microscope (LeicaDMR, Microsystems S.r.l, Wetzlar, Germany). Only clones containing more than 50 cells were considered and counted. For counting, each well was divided into four quadrants, and the media of the number of clones in each quadrant was estimated. The total number of clones per well was then obtained.

### Annexin V/PI apoptosis detection assay

Annexin V/PI apoptosis detection assay (APC Annexin V Apoptosis Detection Kit with PI, cat. n° 640932, BioLegend^®^) was used to identify early and late apoptotic cells. LncH19-silenced HCT-116 cells and respective control cells were seeded at 1.87 × 10^4^ per cm^2^, allowed to adhere overnight, and then treated with 1 µM ITF2357 for 48 h.

Briefly, following the manufacturer’s instructions, cells were harvested, and after centrifugation, cell pellets were washed twice with the cold BioLegend cell staining buffer (Cat. n° 420201), resuspended in annexin V binding buffer, and labeled with APC annexin V and propidium iodide.

Approximately 50,000 events were acquired for each sample on a FACSCanto cytometer (Becton Dickinson, Franklin Lakes, NJ, United States). Flow cytometry data were analyzed using FlowJo software (v10; TreeStar, Ashland, OR, United States).

### Western blotting

H19-silenced HCT-116 cells and control HCT-116 cells were lysed using a lysis buffer (15 mM Tris/HCl pH 7.5, 120 mM NaCl, 25 mM KCl, 1 mM EDTA, and 0.5% Triton X-100) supplemented with phosphatase inhibitor cocktail (Cat. N° 37492, Active Motif, United States) for 1.30 h on ice. Cell debris was removed by centrifugation at 17,000 × g for 15 min at 4°C, and the supernatant, containing the protein lysate, was quantified using the Bradford assay method (Pierce™ Coomassie Plus Assay Kit, cat. N° 23236, Thermo Fisher Scientific, United States) using bovine serum albumin (BSA, cat. n° A2153, Sigma-Aldrich, United States) as a standard. A measure of 15 µg of protein from each sample was separated using Bolt Bis–Tris gel 4%–12% (Cat. n° NP0326BOX, Thermo Fisher Scientific, United States) and transferred onto a nitrocellulose blotting membrane (Amersham Protran Premium 0.45 µm NC by GE Healthcare Life Science, United Kingdom). The membranes were stained with 0.1% red Ponceau in 5% acetic acid to evaluate the correct loading of all samples. The membranes were incubated for 1 h in a blocking solution (5% milk or 5% BSA in 20 mM Tris, 140 mM NaCl, and 0.1% Tween-20) and at 4°C overnight with the following primary antibodies: anti-SQSTM1/p62 (1:500, cat. n° 39749S, Cell Signaling Technology, United States), anti-LC3B (1:500, cat. n° 2775S, Cell Signaling Technology, United States), anti-poly ADP-ribose polymerase-1 (Anti-PARP-1, 1:500, cat. n° sc-8007, Santa Cruz Biotechnology, United States), anti-cleaved caspase-3 (1:400, cat. n° 9664S, Cell Signaling Technology, United States), and anti-p53 (DO-1, 1:200, cat. n° sc-126, Santa Cruz Biotechnology, United States).

After washing with Tris-buffered saline-Tween-20 (TBS-T, 20 mM Tris, 140 mM NaCl, 0.1% Tween-20) three times, the membrane was incubated with appropriate secondary antibodies such as HRP-conjugated goat anti-rabbit IgG (1:10.000, cat. n° 31460, Invitrogen™, Thermo Fisher^®^ Scientific, United States) and anti-mouse IgG (1:10.000, cat. n° 7076, Cell Signaling Technology, United States) at room temperature for 1 h. The chemiluminescent signal was visualized using a chemiluminescence solution (ECL™ Prime Western Blotting System, Cytiva, RPN2232) and detected using the ChemiDoc acquisition instrument (Bio-Rad, United States). The images were analyzed using Image Lab software (Bio-Rad, United States).

Depending on the molecular weight of the protein, if required, the membranes were subjected to a stripping protocol before proceeding with further incubation with other antibodies. This involved a brief incubation of 10–15°min with a stripping solution (Restore™ PLUS Western Blot Stripping Buffer, Cat. n° 46,430, Thermo Fisher^®^ Scientific, United States) at 37°C, followed by subsequent washes in TBS-T.

### LC3-B assay

HCT-116 cells were seeded at 5 × 10^4^ cells/cm^2^ in cell culture chamber slides (Cat. n° 94.6190.802, Sarstedt, Germany), and the LC3B assay (Cat. n°L10382, LC3B Antibody Kit for Autophagy, Invitrogen™ by Thermo Fisher^®^ Scientific, United States) was performed following the manufacturer’s instructions.

Briefly, 24 h after seeding, HCT-116 cells were treated for 24 h with 50 µM chloroquine diphosphate (CQ, provided by the LC3B Antibody Kit for Autophagy) alone or co-treated with 50 µM chloroquine and 1 µM of ITF2357. Chloroquine blocks autophagosome–lysosome fusion, thus allowing autophagosome visualization. After treatments, cells were fixed with 4% paraformaldehyde for 15 min, permeabilized with 0.1% Triton X-100 for 15 min, and incubated with diluted LC3B rabbit polyclonal primary antibody (0.5 μg/mL according to the manufacturer’s instructions) for 1 h. DyLight™ 594 was used as a secondary antibody (Goat anti-Rabbit IgG Secondary Antibody, DyLight™ 594, 1:300, cat n°35560, Invitrogen™ by Thermo Fisher Scientific, United States).

Finally, cells have been counterstained with Hoechst (Hoechst 33342, trihydrochloride, trihydrate, 1:1000, cat n°H3570, Molecular Probes, Life Technologies by Thermo Fisher Scientific, United States) and ActinGreen (ActinGreen™ 488 ReadyProbes™ Reagent, 1:125, cat n°R37110, Invitrogen™ by Thermo Fisher Scientific, United States). All steps have been performed at room temperature. The samples were analyzed using a Nikon A1 confocal microscope.

### RNA extraction and real-time PCR (qRT-PCR)

Total RNA was extracted using the commercially available Macherey–Nagel™ NucleoSpin™ miRNA Kit (Cat. n°740971.250, Macherey–Nagel, Germany), according to the manufacturer’s instructions. The total RNA concentration was detected with the Nanodrop spectrophotometer (Thermo Fisher^®^, United States) and reverse-transcribed to cDNA using the High-Capacity cDNA Reverse Transcription kit (Cat. n° 4368814, Applied Biosystem™, United States).

Quantitative real-time polymerase chain reactions (qRT-PCR) were carried out using the SYBR™ Green PCR Master Mix (Cat. n° 4309155, Applied Biosystems™, United States), following the manufacturer’s instructions in a Step One™ Real-time PCR System Thermal Cycling Block (Applied Biosystems, Waltham, MA, United States).

The primers’ sequences used for expression analysis of the genes of interest are reported in Table 1. Gene expression levels were normalized using β-actin as an endogenous control. Finally, the data are presented as 2^^-ΔΔCt^ compared with the untreated control.

**TABLE 1:** Primers’ sequences of the genes analyzed.

Primer	Forward	Reverse
H19	TCG​TGC​AGA​CAG​GGC​GAC​ATC	CCA​GCT​GCC​ACG​TCC​TGT​AAC​C
SQSTM1/p62	TGT​GTA​GCG​TCT​GCG​AGG​GAA​A	AGT​GTC​CGT​GTT​TCA​CCT​TCC​G
MAP1LC3A	GCT​ACA​AGG​GTG​AGA​AGC​AGC​T	CTG​GTT​CAC​CAG​CAG​GAA​GAA​G
ATG16L	CTA​CGG​AAG​AGA​ACC​AGG​AGC​T	CTG​GTA​GAG​GTT​CCT​TTG​CTG​C
LAMP1	CGT​GTC​ACG​AAG​GCG​TTT​TCA​G	CTG​TTC​TCG​TCC​AGC​AGA​CAC​T
LAMP2	GGC​AAT​GAT​ACT​TGT​CTG​CTG​GC	GTA​GAG​CAG​TGT​GAG​AAC​GGC​A
TP53	CCT​GGA​TTG​GCC​AGA​CTG​C	TTT​TCA​GGA​AGT​AGT​TTC​CAT​AGG​T
NOXA	AGC​TGG​AAG​TCG​AGT​GTG​CT	ACG​TGC​ACC​TCC​TGA​GAA​AA
PUMA	GGAGCAGCACCTGGAGTC	TAC​TGT​GCG​TTG​AGG​TCG​TC
β-ACTIN	TCC​CTT​GCC​ATC​CTA​AAA​GCC​ACC​C	CTG​GGC​CAT​TCT​CCT​TAG​AGA​GAA​G

### Bioinformatic analysis

For predicting interactions between ncRNAs and their targets, bioinformatic analyses were performed using DIANA tools (Rincon-Riveros et al., 2021). Specifically, lncH19–miRNA interactions were identified using DIANA-LncBase v.3, while miRNA–TP53 interactions were identified using DIANA-TarBase v.8.

In *Homo sapiens*, we identified 159 validated miRNAs that lncH19 directly binds to and 42 validated miRNAs that directly bind to the TP53 gene.

By overlaying the two datasets from DIANA-LncBase v.3 (lncH19–miRNA interactions) and DIANA-TarBase v.8 (miRNA–TP53 interactions), we found that lncH19 can bind to 26 miRNAs that directly target the pro-apoptotic TP53 gene (Table 2).

**TABLE 2:** Twenty six miRNAs sponged from lncH19 that directly target the pro-apoptotic TP53 gene.

hsa-let-7a-5p	hsa-miR-17-5p	hsa-miR-107
hsa-let-7b-5p	hsa-miR-19a-3p	hsa-miR-125b-5p
hsa-let-7c-5p	hsa-miR-19b-3p	hsa-miR-181a-5p
hsa-let-7d-5p	hsa-miR-22-3p	hsa-miR-218-5p
hsa-let-7e-5p	hsa-miR-24-3p	hsa-miR-522-5p
hsa-let-7f-5p	hsa-miR-30a-5p	hsa-miR-940
hsa-let-7g-5p	hsa-miR-34a-5p	—
hsa-let-7i-5p	hsa-miR-93-5p	—
hsa-miR-10b-5p	hsa-miR-98-5p	—
hsa-miR-15a-5p	hsa-miR-103a-3p	—

### Statistical analysis

Data reported in all graphs are expressed as the mean ± standard deviation (SD) of at least three independent biological replicates. The following tests have been performed: Student’s t-test to compare two groups, one-way ANOVA for comparisons among three or more groups, and two-way ANOVA for comparison of multiple variables among two groups. Analyses were performed using GraphPad Prism software (GraphPad Software, United States).

*p*-values were indicated in the graphs as follows: * = *p* < 0.05; ** = *p* < 0.01; *** = *p* < 0.001; and **** = *p* < 0.0001. A *p*-value ≤0.05 was considered significant.

## Results

### ITF2357 reduces CRC cell viability and increases the expression levels of lncH19

**FIGURE 1:**
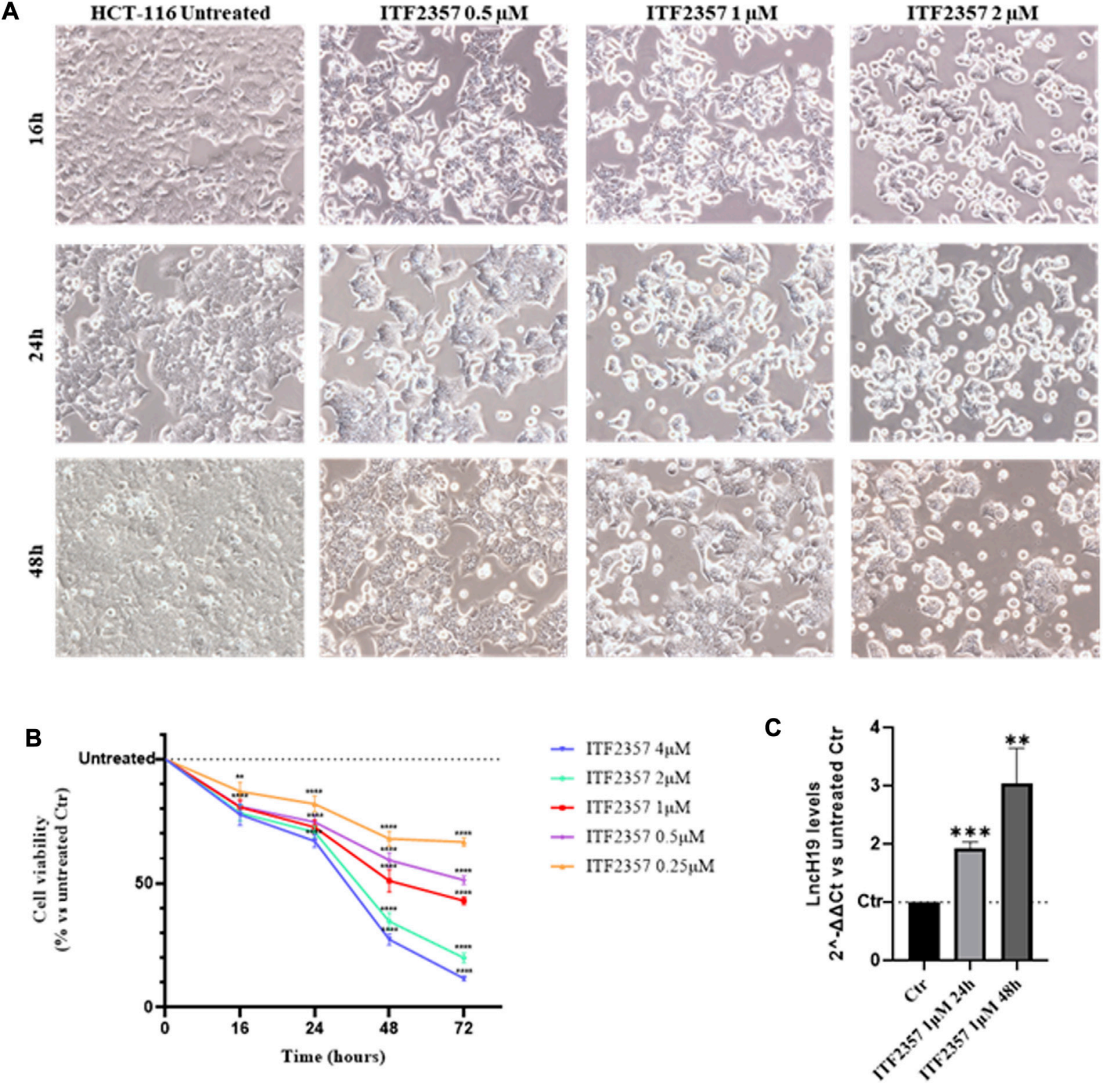
Effects of ITF2357 on HCT-116 cell viability and lncH19 expression. **(A)** Phase contrast images of HCT-116 cells treated with different concentrations of ITF2357 (0.5–1 µM and 2 µM) for 16 h, 24 h, and 48 h. The cells were visualized under a light microscope at ×20 magnification, and the pictures were acquired using NISA1 Leica software. **(B)** Cell viability assay (MTT assay) in HCT-116 cells treated with different concentrations of ITF2357 (0.25–0.5–1–2 µM and 4 µM) for 16 h, 24 h, 48 h, and 72 h. Data are expressed as cell viability percentages compared to untreated cells (Ctr). The results reported in the graph are expressed as the mean ± SD of three independent biological replicates. Statistical analyses were performed using ordinary two-way ANOVA with Bonferroni’s multiple comparison test; **p < 0.01 and ****p < 0.0001. **(C)** Analysis of the expression level (qRT-PCR) of lncH19 in HCT-116 cells treated with 1 µM ITF2357 for 24 h and 48 h. LncH19 expression levels are reported as 2^^-ΔΔCt^ compared to untreated cells (Ctr), and the threshold cycle (Ct) was normalized against β-actin. The results reported in the graph are expressed as the mean ± SD of three independent biological replicates. Statistical analyses were performed using Student’s t-test; *p < 0.05 and **p < 0.01.

Initially, to evaluate the sensitivity of the HCT-116 CRC cell line to ITF2357, cells were treated with different concentrations of ITF2357 for 16 h, 24 h, 48 h, and 72 h. Evaluation of cell morphology indicated that the drug exerted a cytotoxic effect, which appeared after 24 h in cells treated with 1 μM ITF2357 and was clearly evident after 48 h either with 1 μM or 2 μM (Figure 1A). Morphological data were confirmed by the MTT assay (Figure 1B). As expected, ITF2357 treatment reduced the viability of HCT-116 cells in a dose- and time-dependent manner. Approximately 50% reduction in viability was observed after 48 h of treatment with 1 μM ITF2357.

LncH19 is known to display the oncogenic activity in CRC, promoting cell proliferation (Yang et al., 2017), epithelial-to-mesenchymal transition (Ding et al., 2018), and 5-FU drug resistance (Wang et al., 2018). To elucidate whether HDACi modifies the expression levels of lncH19, we performed qRT-PCR analyses. Interestingly, the results revealed that ITF2357 promoted lncH19 expression in HCT-116, determining a two-fold increase in the level of lncRNA after 24 h of treatment and almost three-fold increase at 48 h (Figure 1C). Therefore, we hypothesized that lncH19 induction could somehow be functional to ITF2357 to exert its cytotoxic effect.

**FIGURE 2:**
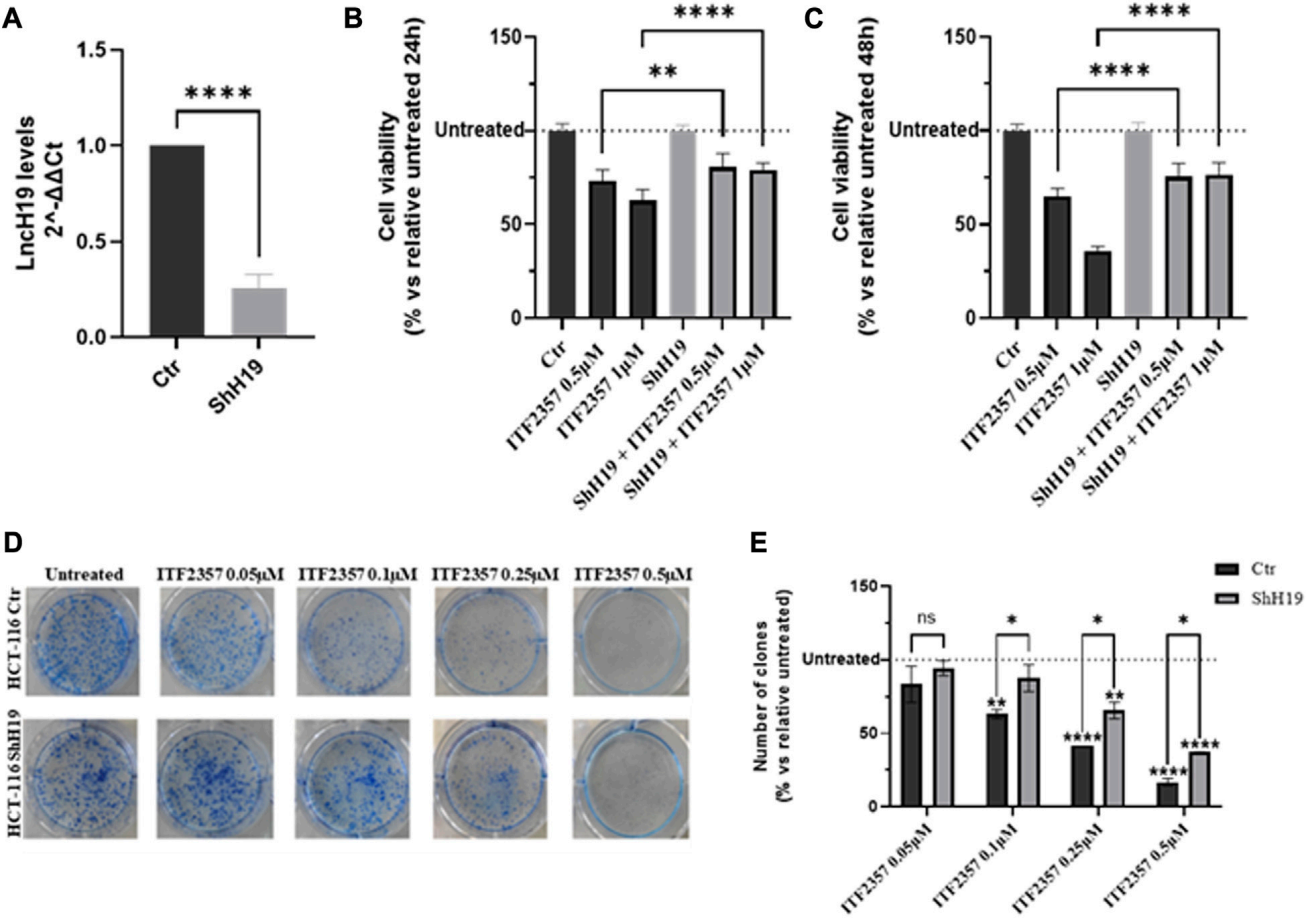
Effects of silencing lncH19 in HCT-116-silenced cells treated with ITF2357. **(A)** Analysis of the expression level (qRT-PCR) of lncH19 in HCT-116-silenced cells with respect to control cells (Ctr). LncH19 expression levels are reported as 2^^-ΔΔCt^ compared to control cells (Ctr); Ct was normalized against β-actin. Data are expressed as the mean ± SD of three independent biological replicates. Statistical analyses were performed using Student’s t-test, ****p < 0.0001. **(B, C)** Cell viability assay (MTT assay) in HCT-116 cells (that are silenced or not) for lncH19 and treated with two different concentrations of ITF2357 (0.5 and 1 µM) for 24 h (left graph) and 48 h (right graph). Data are expressed as the cell viability percentage compared to untreated cells. Data are expressed as the mean ± SD of three independent biological replicates. Statistical analyses were performed using ordinary one-way ANOVA with Bonferroni’s multiple comparison test; **p < 0.01 and ****p < 0.0001. **(D, E)** Clonogenic assay in HCT-116 cells with silenced or unsilenced lncH19 cells, untreated or treated with indicated concentrations of ITF2357, and maintained in culture for 8 days to allow clone formation. In the histogram, data are expressed as a percentage of the number of clones compared to relative untreated cells. Data are expressed as the mean ± SD. Statistical analyses were performed using ordinary two-way ANOVA with Bonferroni’s multiple comparison test; *p < 0.05, **p < 0.01, ***p < 0.001, and ****p < 0.0001.

To verify this hypothesis, HCT-116 cells were stably silenced for lncH19, and the silencing efficiency was confirmed through gene expression analysis (Figure 2A). Cell viability assays in H19-silenced cells revealed that ITF2357 displayed much less efficacy under lncH19 knockdown. Indeed, the effect of ITF2357 was reduced by approximately 15%, suggesting that lncH19 plays a role in ITF2357-induced cytotoxicity in CRC cells (Figures 2B, C).

Moreover, colony formation assay further confirmed a direct role of lncH19 to sustain the efficacy of HDACi in CRC cells. Specifically, as shown in Figure 2D, treatment with ITF2357 affected the clonogenicity of HCT-116 control cells in a dose-dependent manner, while this effect was significantly weaker in H19-silenced cells, as also revealed by the quantification of the number of clones in the two cell types (Figure 2E).

### ITF2357 induces pro-survival autophagy in CRC cells

**FIGURE 3:**
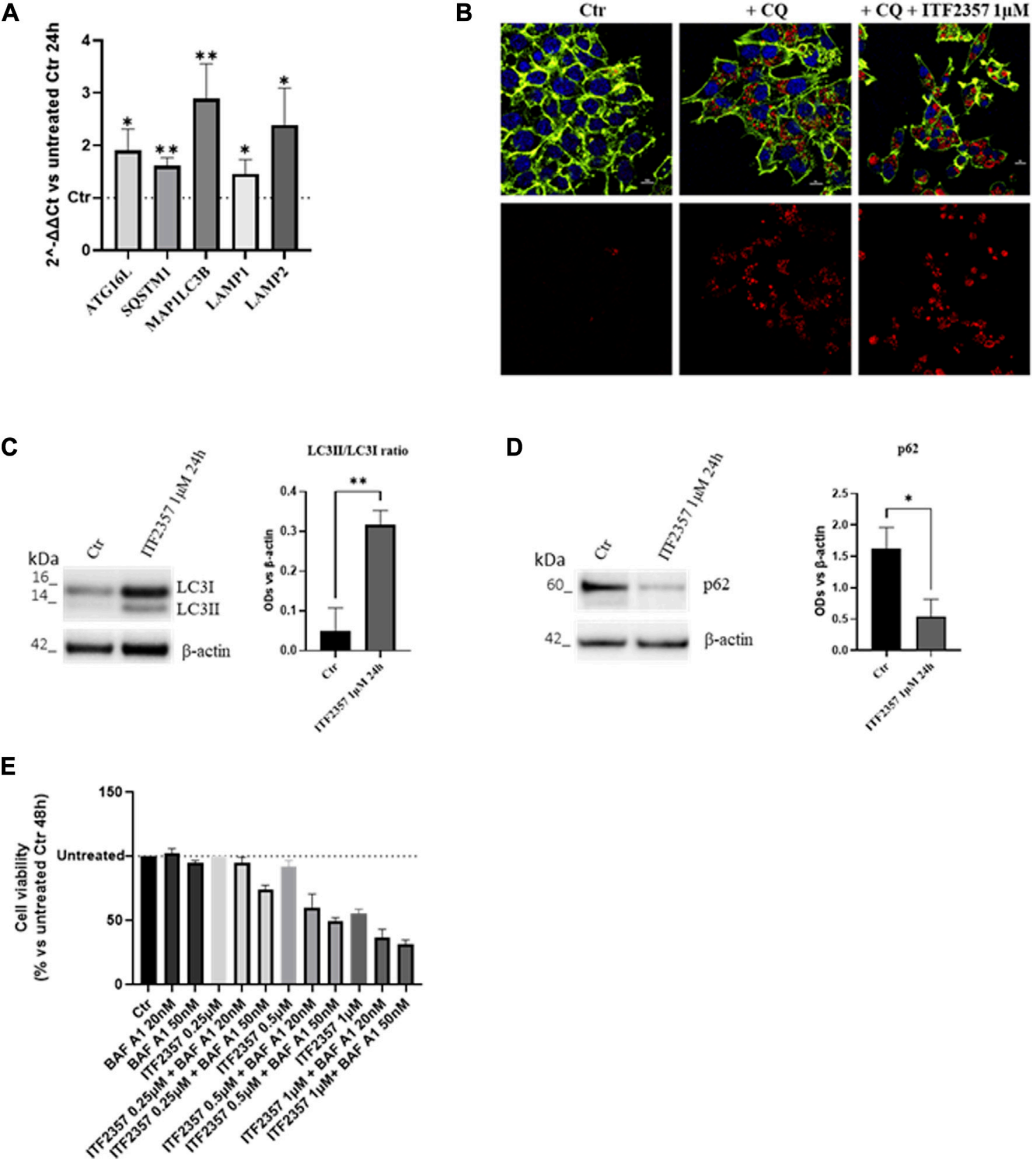
HDAC inhibitor ITF2357 induces survival autophagy in CRC cells. **(A)** Analysis of the expression level (qRT-PCR) of autophagic genes in HCT-116 cells treated with 1 µM concentration of ITF2357 for 24 h. The expression levels of genes are reported as 2^^-ΔΔCt^ compared to untreated cells (Ctr), and Ct was normalized against β-actin. Data are expressed as the mean ± SD of three independent biological replicates. Statistical analyses were performed using Student’s t-test; *p < 0.05 and **p < 0.01. **(B)** Immunofluorescence for LC3B on HCT-116 cells, untreated or treated with 50 µM chloroquine diphosphate (CQ) alone or in combination with 1 µM of ITF2357 for 24 h. LC3B is represented in red, counterstained with Hoechst and ActinGreen, for nuclei in blue and cytoskeleton in green, respectively. Nuclear focal plane; the scale bar is 10 µm. **(C)** Representative images and densitometric analysis of Western blots for LC3II/LC3I in HCT-116 cells treated or not with ITF2357 1 µM for 24 h. The graph shows the ratio of the normalized optical density (OD). Housekeeping β-actin was used as a loading control. Data are expressed as the mean ± SD of three independent biological replicates. Statistical analyses were performed using Student’s t-test, **p < 0.01. **(D)** Representative images and densitometric analysis of Western blots for p62 in cells treated or not with ITF2357 1 mM concentration for 24 h. The graph shows the normalized OD. Housekeeping β-actin was used as a loading control. Data are expressed as the mean ± SD of three independent biological replicates. Statistical analyses were performed using Student’s t-test, *p < 0.05. **(E)** Cell viability assay (MTT assay) in HCT-116 cells co-treated with different concentrations of ITF2357 (0.25–0.5 µM and 1 µM) and two different concentrations of bafilomycin A1 (20 nM and 50 nM) for 48 h. Data are expressed as cell viability percentages compared to untreated cells (Ctr). Data are expressed as the mean ± SD.

It is well known that both HDACis and lncH19 induce autophagy in different tumor cells (Xu et al., 2018; Mrakovcic and Frohlich, 2019; Zhao et al., 2021). Therefore, we hypothesized that ITF2357, enforced by H19 expression, induces autophagy-dependent cell death. To verify this hypothesis, the transcriptional levels of some autophagy markers (ATG16L, SQSTM1/p62, MAP1LC3B/LC3, and LAMP1/2) were analyzed. As shown in Figure 3A, ITF2357 upregulated all the autophagy genes analyzed, an effect that was already evident after 24 h. This effect was maintained after 48 h of treatment (data not shown).

The activation of autophagy was confirmed by an increase in the LC3B signal in autophagosomes, as revealed by immunofluorescence (Figure 3B). These data were confirmed by Western blot analysis, showing a much higher level of LC3II-cleaved form in ITF2357-treated cells. Moreover, further confirmation of the autophagic process induced by ITF2357 was sustained by the significant decrease in the levels of p62 protein (Figures 3C, D). This marker is usually considered to monitor the autophagic flux, and it is associated with completed autophagy when decreasing since it is degraded by the autophagosome (Emanuele et al., 2020).

To investigate whether the activation of autophagy in HCT-116 cells could promote cell death, cell viability was evaluated in cells treated with ITF2357 in the presence of the autophagy inhibitor bafilomycin A1.

As shown in Figure 3E, the cytotoxic effect exerted by three different doses of ITF2357 was enhanced when co-treated with either 20 nM or 50 nM bafilomycin A1. These data suggest that autophagy induced by the HDAC inhibitor represents a pro-survival adaptive response to the effects of the compound. Moreover, we provided evidence that H19 silencing did not affect ITF2357-induced autophagy (Supplementary Figure S1).

### ITF2357 induces apoptosis in HCT-116 cells, and lncH19 is functional to this effect

**FIGURE 4:**
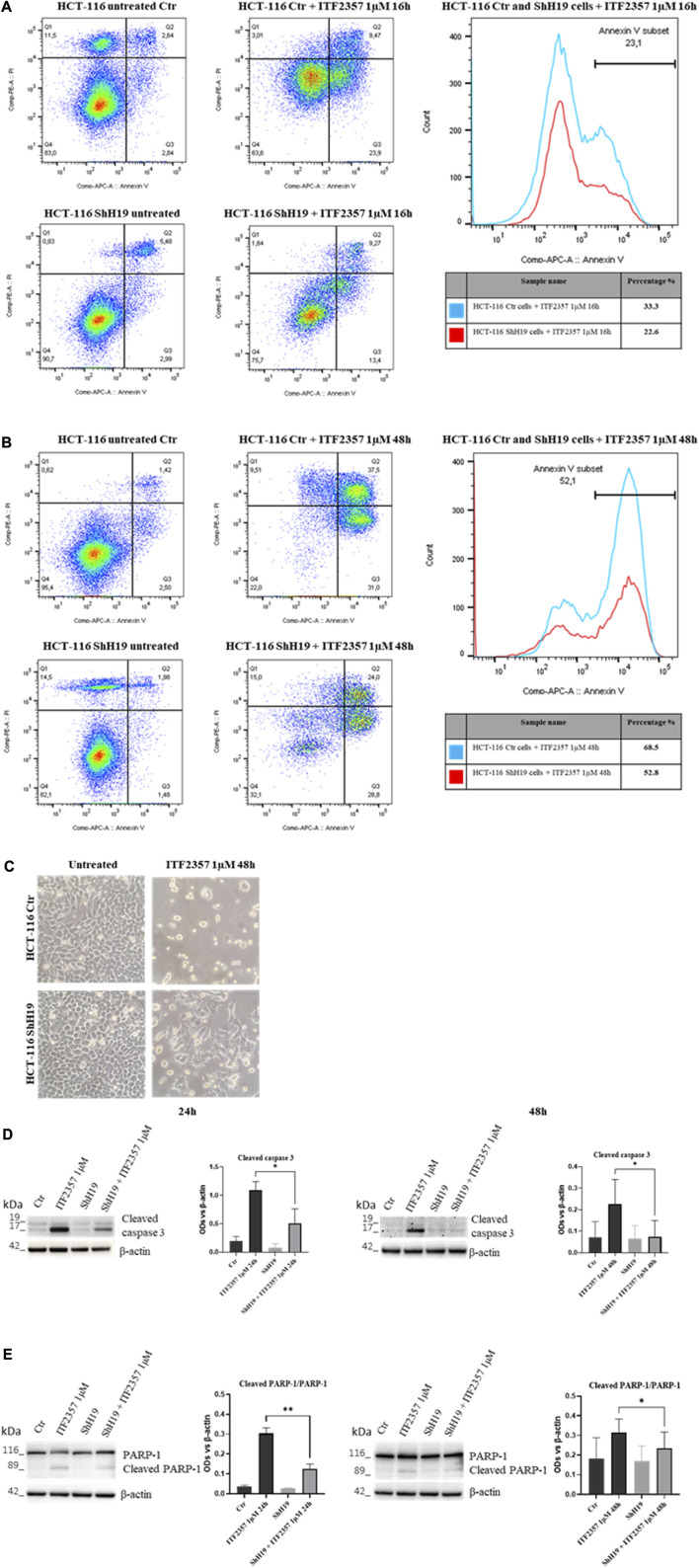
(Continued).

To further characterize cell death activated in response to ITF2357 and elucidate the role of lncH19, apoptosis was investigated in H19-silenced cells in comparison with the respective control cells. Specifically, an annexin V/PI apoptotic assay was performed at early (16 h) and late (48 h) treatment time points to properly detect the process over time. The results shown in Figures 4A, B indicate that ITF2357 stimulated early and late apoptosis to a different extent in control and H19-silenced cells. Indeed, the total percentage of annexin V positive cells after treatment with ITF2357 was approximately 33% in control cells, compared to 22.6% in H19-silenced cells at 16 h. Such a difference was maintained at 48 h (68.6% in control cells vs. 52.8% in H19-silenced cells), thus confirming that lncH19 knockdown reduces the apoptotic efficacy of ITF2357.

Morphological analysis of ITF2357-treated cells clearly showed the differential effect of HDACi in the two cell types (Figure 4C).

These data were confirmed by Western blot analysis of apoptotic markers, including cleaved caspase 3 and cleaved PARP-1, an analysis that was performed at late time points to evidence apoptosis execution. As shown in Figures 4D, E, although caspase 3 cleavage and PARP-1 degradation were evident in ITF2357-treated control cells, these effects were much less evident in H19-silenced cells. These data suggest that H19 expression somehow reinforces the pro-apoptotic action of ITF2357.

**FIGURE 5:**
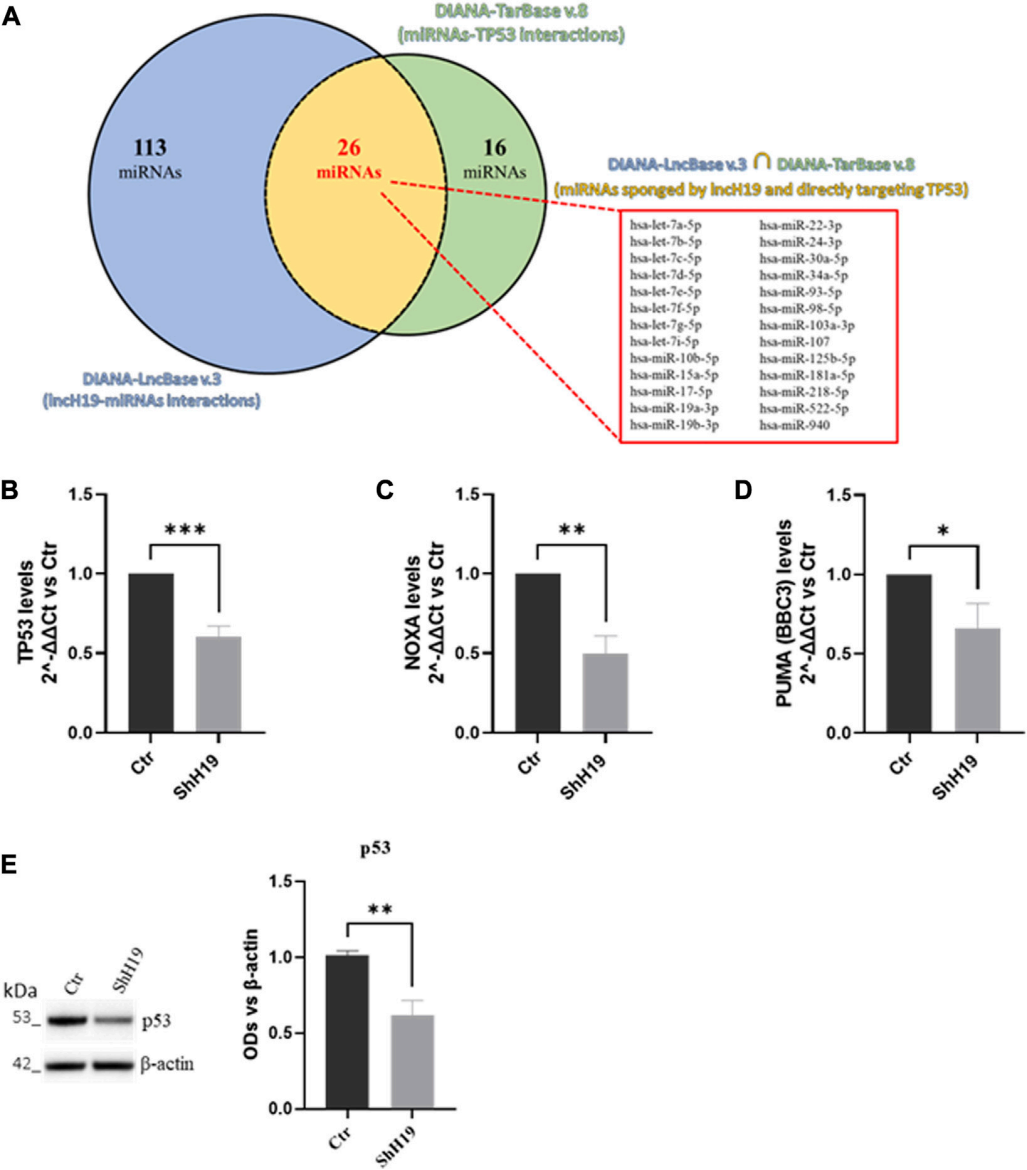
Identification of lncH19 miRNAs that target TP53. **(A)** Venn diagram obtained by bioinformatic analysis using DIANA tools, illustrating the intersection (in yellow) between the dataset of validated direct miRNAs that lncH19 binds to (DIANA-LncBase v.3, in blue) and the dataset of validated miRNAs that directly bind to TP53 (DIANA-TarBase v.8, in green). The intersection shows 26 miRNAs (listed in the panel) sponged from lncH19 that directly target the pro-apoptotic TP53 gene. **(B–D)** Analysis of the expression levels (qRT-PCR) of TP53 **(B)**, NOXA **(C),** and PUMA **(D)** in HCT-116 cells with respect to control cells (Ctr). Gene expression levels are reported as 2^^-ΔΔCt^ compared to control cells (Ctr); Ct was normalized against β-actin. Data are expressed as the mean ± SD of three independent biological replicates. Statistical analyses were performed using Student’s t-test; *p < 0.05, **p < 0.01, and ***p < 0.001. **(E)** Representative images and densitometric analysis of Western blots for p53 in HCT-116 with respect to control cells (Ctr). The graphs show the OD of the indicated proteins normalized for the housekeeping’s OD(β-actin). Data are expressed as the mean ± SD of three independent biological replicates. Statistical analyses were performed using Student’s t-test, **p < 0.01.

To investigate the molecular mechanism by which lncH19 promotes ITF2357-induced apoptosis, we focused on identifying putative miRNAs with a pro-apoptotic role that could be targeted by lncH19. Similar to other lncRNAs, H19 can also behave as an endogenous competitive sponge for miRNAs (Ye et al., 2019). By using DIANA tools (Rincon-Riveros et al., 2021), we identified 159 validated human miRNAs sponged by lncH19, and among these, 26 validated human miRNAs directly target the pro-apoptotic TP53 gene (Figure 5A). Real-time PCR in Figure 5B confirmed a positive correlation between the expression of lncH19 and TP53. The transcriptional analyses revealed that cells silenced for lncH19 express lower levels of TP53 and its targets, PUMA and NOXA (Figures 5B–D). The reduction of p53 in shH19 cells was further confirmed at the protein level (Figure 5E). Overall, these data indicate that ITF2357 induces TP53-mediated apoptosis in colorectal cancer cells, and the expression of lncH19 plays a functional role in regulating p53 expression.

**FIGURE 6:**
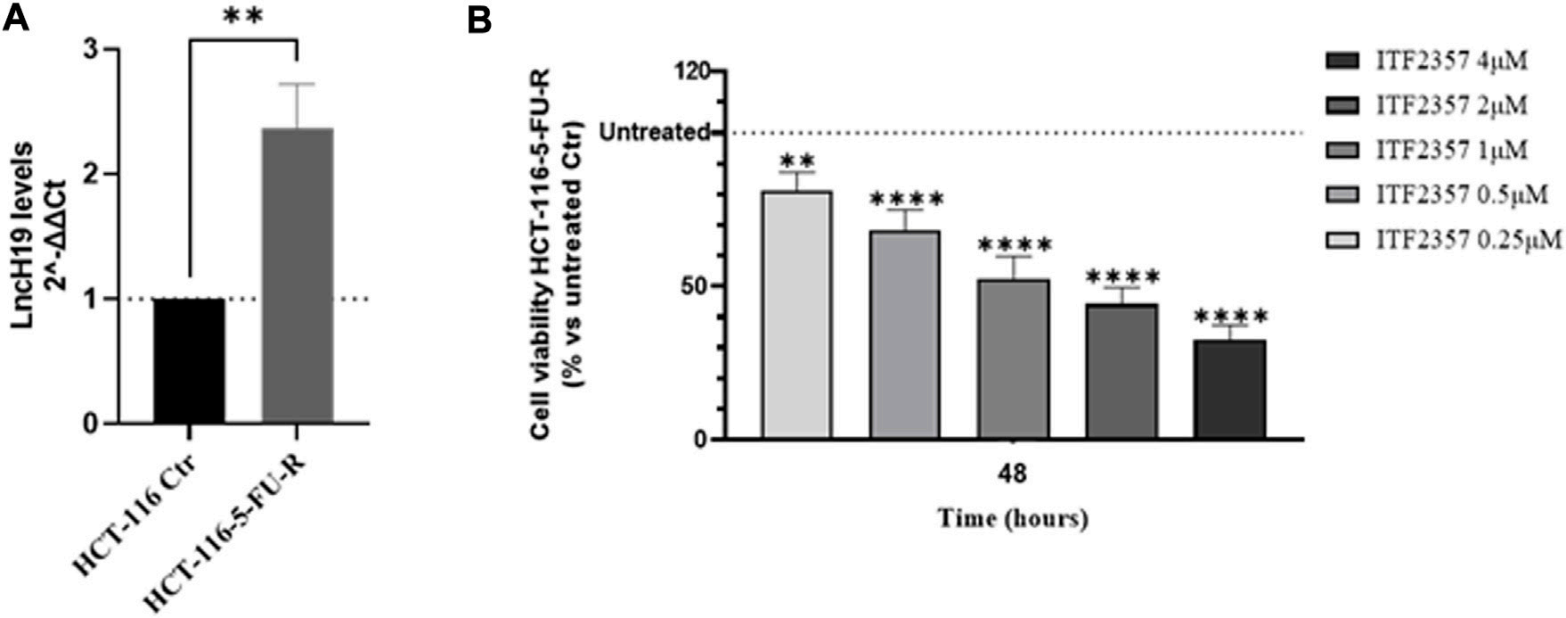
HCT-116 cells resistant to 5-fluorouracil (5-FU) express high levels of lncH19 and respond to treatment with ITF2357. **(A)** Analysis of the expression level (qRT-PCR) of lncH19 in HCT-116-5-FU-R cells compared to untreated cells (HCT-116 Ctr). LncH19 expression levels are reported as 2^^-ΔΔCt^ compared to HCT-116 Ctr cells, and Ct was normalized against β-actin. The results reported in the graph are expressed as the mean ± SD of three independent biological replicates. Statistical analyses were performed using Student’s t-test, **p < 0.01. **(B)** Cell viability assay (MTT assay) in HCT-116-5-FU-R cells treated with different concentrations of ITF2357 (0.25–0.5–1–2 µM and 4 µM) for 48 h. Data are expressed as cell viability percentages compared to untreated cells (Ctr). The results reported in the graph are expressed as the mean ± SD of three independent biological replicates. Statistical analyses were performed using ordinary one-way ANOVA with Bonferroni’s multiple comparison test; **p < 0.01 and ****p < 0.0001.

Finally, to assess whether ITF2357 can overcome the resistance to 5-FU chemotherapeutics, we used the HCT-116-5-FU-R, a 5-FU-resistant HCT-116 cell line properly selected in our laboratory. Interestingly, HCT-116-5-FU-R cells express high levels of lncH19 compared to parental HCT-116 cells (Figure 6A). It is noteworthy that these cells nicely respond to ITF2357, as indicated by the cell viability evaluation reported in Figure 6B, which revealed a dose-dependent effect of the compound.

## Discussion

This paper shows, for the first time, that lncH19 supports apoptosis induced by HDACi ITF2357 in colon cancer cells. Although some papers sustain the potential of HDACis in colon cancer treatment (Garmpis et al., 2022; Lee et al., 2022), to date, no evidence has been provided about the efficacy of this pan-HDACi in colon cancer cells. Our data indicate that ITF2357 is active in colon cancer cells at micromolar concentrations, in line with the findings of other authors in different tumor cell lines (Angeletti et al., 2016; Di Martile et al., 2018; Celesia et al., 2022; Celesia et al., 2023).

We also provided evidence that ITF2357 upregulates lncH19 in colon cancer cells. Similarly, Di Fazio et al. found increased lncH19 levels in adrenocortical carcinoma, following treatment with pan-HDACis such as panobinostat, trichostatin A (TSA), and SAHA, correlated with autophagy induction (Di Fazio et al., 2022).

To understand the role of lncH19 in ITF2357-induced cytotoxicity in colon cancer cells, both autophagy and apoptosis induction were examined in H19 stably silenced HCT-116 cells in comparison with control HCT-116 cells. It is well known that HDACis can promote autophagy in different tumor types (Bai et al., 2019; Xiao et al., 2020; Korholz et al., 2021). However, it is well known that autophagy can exert a dual role in tumor cells. Indeed, the process can be activated as a pro-survival response, which is frequently associated with tumor progression and chemoresistance, or it can serve a death-inducing function, thereby representing an alternative form of cell death to target tumor cells that have developed apoptosis resistance (Patra et al., 2019). This paper shows that ITF2357 promoted the expression of autophagy markers, including ATG16L, SQSTM1/p62, MAP1LC3B/LC3, and LAMP1/2. HDACi also induced the conversion of LC3I into active LC3II and a reduction in the levels of p62. Our data support the hypothesis that ITF2357-induced autophagy is correlated with a pro-survival cell response since the autophagy inhibitor bafilomycin A1 markedly potentiated the cytotoxic effect of the compound and the p62 protein marker decreased, indicating autophagy completion (Emanuele et al., 2020). Our findings are in accordance with the observation of Angeletti et al., who found that inhibition of autophagy potentiates the effect of ITF2357 in glioblastoma cells (Angeletti et al., 2016). However, our Supplementary Material indicates that lncH19 silencing does not significantly modify the levels of autophagy markers.

Therefore, we concluded that the cytotoxic effect of ITF2357 does not depend on autophagy-induced cell death, and subsequently, caspase-dependent apoptosis was considered.

Evaluation of apoptosis by annexin V/PI double staining and analysis of apoptotic markers revealed that lncH19 plays a role in this event. Indeed, ITF2357-induced apoptosis was reduced in H19-silenced cells compared to the respective control cells. We consider these results relevant since they imply that lncH19 can be exploited to favor apoptosis induction and that HDACi may promote a H19-dependent targeted effect in colon cancer cells. In accordance with our results, other authors have previously found a correlation between lncH19 and apoptosis.

In particular, Hou et al. have shown that overexpressed lncH19 alleviates induced lung injury in mice, as well as lipopolysaccharide (LPS)-induced apoptosis, oxidative stress, and inflammation (Hou et al., 2022). Similarly, Yang provided evidence that H19 silencing alleviates LPS-induced apoptosis and inflammation by regulating the miR-140-5p/TLR4 axis in cell models of pneumonia (Yang, 2023). In a more specific tumoral context, lncH19 has been shown to participate in triptolide/TNF-α-induced apoptosis via binding miR-204-5p in gastric cancer models (Yuan et al., 2022). In addition, Liu et al. demonstrated that lncH19 inhibits proliferation and enhances apoptosis of nephroblastoma cells by regulating the miR-675/TGFBI axis (Liu et al., 2022). Accordingly, lncH19 has also been implicated in sensitization to X-ray and carbon ion irradiation of non-small cell lung cancer (Zhao et al., 2021), and positively modulates the sensitivity of glioma cells to radiation-favoring apoptosis (Kuang et al., 2021). However, some controversial data are present in the literature regarding the pro-apoptotic role of lncH19. For instance, the knockdown of H19 in resveratrol-treated cancer cells has been shown to enhance the effects of resveratrol on apoptosis (Li et al., 2022). Other evidence of an antiapoptotic role of lncH19 was provided by Wang et al., who showed that it promotes proliferation, migration, and invasion, and inhibits apoptosis of breast cancer cells by targeting the miR-491-5p/ZNF703 axis (Wang et al., 2020). It is clear that lncRNA H19 and many other cellular factors may exert a dual role in regulating cell fate (Shermane Lim et al., 2021).

**FIGURE 7:**
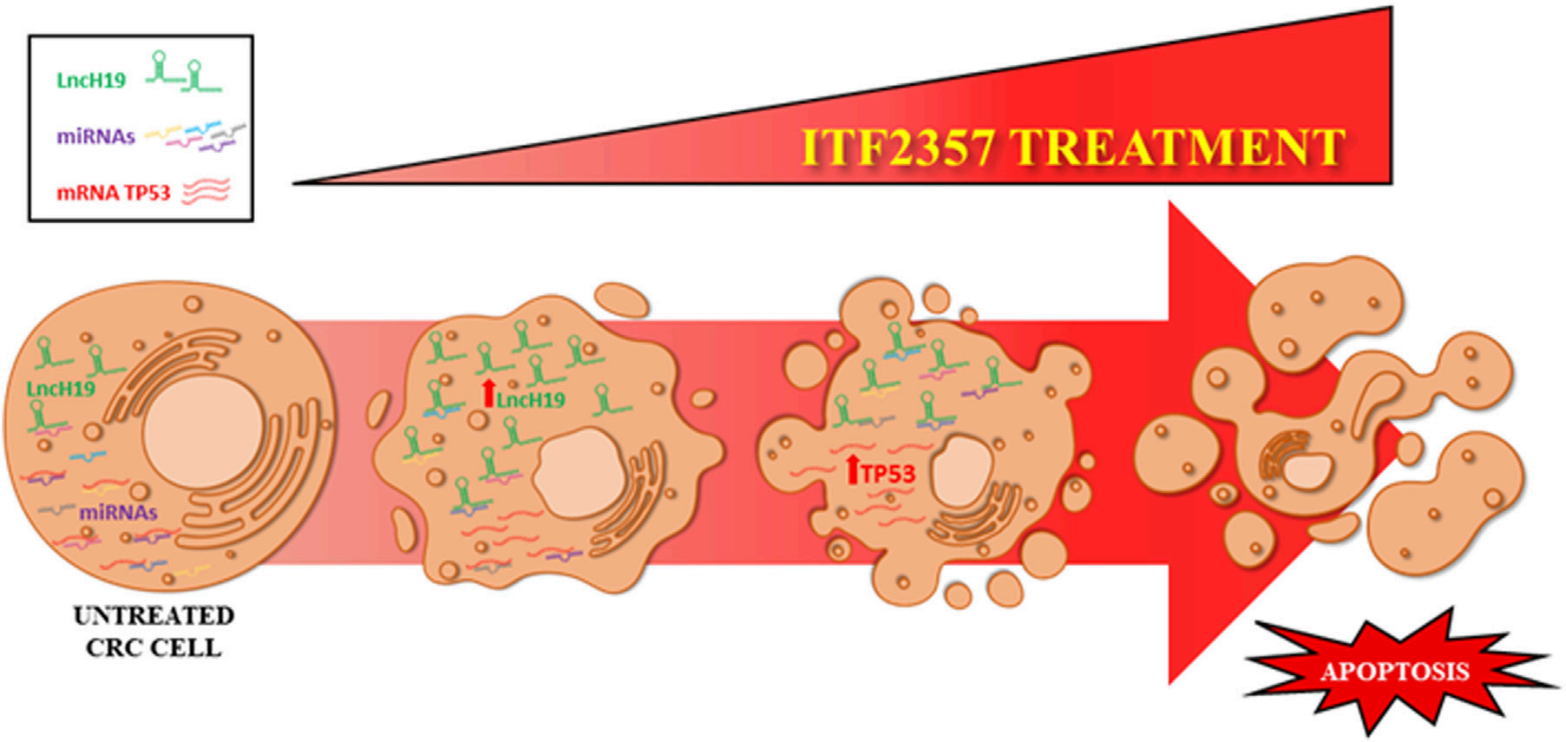
Schematic representation of the proposed model. The levels of lncH19 increase in CRC cells treated with HDACi ITF2357. This increases the sponge effect by lncH19 on miRNAs targeting pro-apoptotic genes, including TP53. Overall, treatment with ITF2357 increases lncH19 levels and promotes activation of apoptosis, thus leading to increased expression of TP53.

Our data strongly suggest a pro-apoptotic role of lncH19 in CRC cells treated with HDACi ITF2357 since lncH19 silencing profoundly reduced the effects of the compound on cell viability and apoptosis. To explain the pro-apoptotic role of lncH19 in HDACi-treated cells, we hypothesized that it may act as an endogenous competitive sponge for miRNAs (Zhang et al., 2022), antagonizing miRNAs targeting pro-apoptotic genes. Bioinformatic analysis revealed that lncH19 sponged 26 validated human miRNAs directly targeting the pro-apoptotic gene TP53 (Figure 7).

Our data provide evidence that lncH19 knockdown reduces the expression of TP53 and its pro-apoptotic targets, PUMA and NOXA. The relationship between lncH19 and TP53 is controversial in the literature since some papers sustain a negative control of TP53 by H19 (Yang et al., 2012; Li et al., 2020; Gan et al., 2022), while others support that lncH19 may activate the tumor suppressor. Specifically, in accordance with our findings, we have shown that overexpression of lncH19 enhanced TP53 expression, whereas H19 silencing exerted the opposite effect (Zhuang et al., 2021). In addition, Du et al. have found that lncH19 promotes p53 phosphorylation by a direct interaction, an effect that results in increased NOTCH-mediated angiogenesis in mesenchymal stem cells (Du et al., 2023).

Interestingly, our paper also provided evidence that lncH19 is overexpressed in HCT-116-5-FU-R cells, and we consider it relevant that the HDACi ITF2357 was capable of overcoming 5-FU resistance in these cells. Other authors have associated 5-FU resistance with lncH19 expression (Wang et al., 2018; Yokoyama et al., 2019; Zhang et al., 2022); here, we suggest that this condition may be exploited to promote TP53-dependent apoptosis using HDACi. To date, several lines of evidence indicate that HDACi can sensitize different tumor types to the effects of diverse chemotherapeutic agents (Perego et al., 2012; Almeida et al., 2017; Minegaki et al., 2018; Rodrigues Moita et al., 2020; Roca et al., 2022).

It has to be considered that the present study refers to CRC cell lines, with all the limitations to an *in vitro* study; however, it represents a molecular basis to proceed with translational studies. In particular, we provided evidence for the first time that HDACi ITF2357 is efficacious in a colon cancer model by upregulating lncH19 and is capable of overcoming 5-FU resistance in highly H19-expressing CRC cells. These findings need to be validated *in vivo* for a possible clinical application in CRC patients displaying 5-FU drug resistance. In our opinion, the relevant finding was that lncH19, which canonically acts as an oncogene, may be exploited to favor apoptosis induced by ITF2357. This implies that high expression of lncH19 in CRC, especially in conditions of 5-FU resistance, may facilitate apoptosis induction.

Overall, our data suggest that lncH19 levels may be a useful parameter to promote epigenetic targeting of colon cancer and propose ITF2357 as a promising epi-drug in colon cancer treatment.

## Data Availability

The raw data supporting the conclusion of this article will be made available by the authors, without undue reservation.

## References

[B1] Almeida L. O. Guimaraes D. M. Martins M. D. Martins M. A. T. Warner K. A. Nor J. E. (2017). Unlocking the chromatin of adenoid cystic carcinomas using HDAC inhibitors sensitize cancer stem cells to cisplatin and induces tumor senescence. Stem Cell. Res. 21, 94–105. 10.1016/j.scr.2017.04.003 28426972 PMC7071815

[B2] Angeletti F. Fossati G. Pattarozzi A. Wurth R. Solari A. Daga A. (2016). Inhibition of the autophagy pathway synergistically potentiates the cytotoxic activity of givinostat (ITF2357) on human glioblastoma cancer stem cells. Front. Mol. Neurosci. 9, 107. 10.3389/fnmol.2016.00107 27833530 PMC5081386

[B3] Bai Y. Chen Y. Chen X. Jiang J. Wang X. Wang L. (2019). Trichostatin A activates FOXO1 and induces autophagy in osteosarcoma. Arch. Med. Sci. 15 (1), 204–213. 10.5114/aoms.2018.73860 30697272 PMC6348367

[B4] Bao M. H. Szeto V. Yang B. B. Zhu S. Z. Sun H. S. Feng Z. P. (2018). Long non-coding RNAs in ischemic stroke. Cell. Death Dis. 9 (3), 281. 10.1038/s41419-018-0282-x 29449542 PMC5833768

[B5] Bitarafan S. Yari M. Broumand M. A. Ghaderian S. M. H. Rahimi M. Mirfakhraie R. (2019). Association of increased levels of lncRNA H19 in PBMCs with risk of coronary artery disease. Cell. J. 20 (4), 564–568. 10.22074/cellj.2019.5544 30124004 PMC6099137

[B6] Celesia A. Franzo M. Di Liberto D. Lauricella M. Carlisi D. D'Anneo A. (2023). Oncogenic BRAF and p53 interplay in melanoma cells and the effects of the HDAC inhibitor ITF2357 (givinostat). Int. J. Mol. Sci. 24 (11), 9148. 10.3390/ijms24119148 37298104 PMC10252263

[B7] Celesia A. Notaro A. Franzo M. Lauricella M. D'Anneo A. Carlisi D. (2022). The histone deacetylase inhibitor ITF2357 (givinostat) targets oncogenic BRAF in melanoma cells and promotes a switch from pro-survival autophagy to apoptosis. Biomedicines 10 (8), 1994. 10.3390/biomedicines10081994 36009541 PMC9405675

[B8] Comi G. P. Niks E. H. Vandenborne K. Cinnante C. M. Kan H. E. Willcocks R. J. (2023). Givinostat for becker muscular dystrophy: A randomized, placebo-controlled, double-blind study. Front. Neurol. 14, 1095121. 10.3389/fneur.2023.1095121 36793492 PMC9923355

[B9] Corrado C. Costa V. Giavaresi G. Calabrese A. Conigliaro A. Alessandro R. (2019). Long non coding RNA H19: A new player in hypoxia-induced multiple myeloma cell dissemination. Int. J. Mol. Sci. 20 (4), 801. 10.3390/ijms20040801 30781795 PMC6413127

[B10] Costa V. Lo Dico A. Rizzo A. Rajata F. Tripodi M. Alessandro R. (2017). MiR-675-5p supports hypoxia induced epithelial to mesenchymal transition in colon cancer cells. Oncotarget 8 (15), 24292–24302. 10.18632/oncotarget.14464 28061476 PMC5421847

[B11] Cui J. Xu F. Bai W. Zhao T. Hong J. Zuo W. (2023). HDAC inhibitor ITF2357 reduces resistance of mutant-KRAS non-small cell lung cancer to pemetrexed through a HDAC2/miR-130a-3p-dependent mechanism. J. Transl. Med. 21 (1), 125. 10.1186/s12967-023-03973-3 36793108 PMC9930237

[B12] Dai L. Li J. Dong Z. Liu Y. Chen Y. Chen N. (2019). Temporal expression and functional analysis of long non-coding RNAs in colorectal cancer initiation. J. Cell. Mol. Med. 23 (6), 4127–4138. 10.1111/jcmm.14300 30920116 PMC6533480

[B13] Dasko M. de Pascual-Teresa B. Ortin I. Ramos A. (2022). HDAC inhibitors: Innovative strategies for their design and applications. Molecules 27 (3), 715. 10.3390/molecules27030715 35163980 PMC8837987

[B14] Di Fazio P. Rusche F. D. Roth S. Pehl A. Wachter S. Mintziras I. (2022). Long non-coding RNA H19 expression correlates with autophagy process in adrenocortical carcinoma. Cancer Invest. 40 (3), 254–267. 10.1080/07357907.2021.2001483 34726962

[B15] Di Martile M. Desideri M. Tupone M. G. Buglioni S. Antoniani B. Mastroiorio C. (2018). Histone deacetylase inhibitor ITF2357 leads to apoptosis and enhances doxorubicin cytotoxicity in preclinical models of human sarcoma. Oncogenesis 7 (2), 20. 10.1038/s41389-018-0026-x 29472530 PMC5833676

[B16] Ding D. Li C. Zhao T. Li D. Yang L. Zhang B. (2018). LncRNA H19/miR-29b-3p/PGRN Axis promoted epithelial-mesenchymal transition of colorectal cancer cells by acting on wnt signaling. Mol. Cells 41 (5), 423–435. 10.14348/molcells.2018.2258 29754471 PMC5974619

[B17] Du C. Cheng Q. Zhao P. Wang C. Zhu Z. Wu X. (2023). LncRNA H19 mediates BMP9-induced angiogenesis in mesenchymal stem cells by promoting the p53-Notch1 angiogenic signaling axis. Genes. Dis. 10 (3), 1040–1054. 10.1016/j.gendis.2022.04.013 37396541 PMC10308131

[B18] Eckschlager T. Plch J. Stiborova M. Hrabeta J. (2017). Histone deacetylase inhibitors as anticancer drugs. Int. J. Mol. Sci. 18 (7), 1414. 10.3390/ijms18071414 28671573 PMC5535906

[B19] Emanuele S. Lauricella M. D'Anneo A. Carlisi D. De Blasio A. Di Liberto D. (2020). p62: Friend or foe? Evidences for OncoJanus and NeuroJanus roles. Int. J. Mol. Sci. 21 (14), 5029. 10.3390/ijms21145029 32708719 PMC7404084

[B20] Feng Y. Yang C. Hu D. Wang X. Liu X. (2017). miR-675 promotes disease progression of non-small cell lung cancer via activating NF-κB signaling pathway. Cell. Mol. Biol. (Noisy-le-grand) 63 (5), 7–10. 10.14715/cmb/2017.63.5.2 28719338

[B21] Gan L. Liao S. Tong Y. Li W. Peng W. Deng S. (2022). Long noncoding RNA H19 mediates neural stem/progenitor cells proliferation, differentiation and apoptosis through the p53 signaling pathway after ischemic stroke. Biochem. Biophys. Res. Commun. 597, 8–15. 10.1016/j.bbrc.2022.01.095 35121179

[B22] Garmpis N. Damaskos C. Garmpi A. Nonni A. Georgakopoulou V. E. Antoniou E. (2022). Histone deacetylases and their inhibitors in colorectal cancer therapy: Current evidence and future considerations. Curr. Med. Chem. 29 (17), 2979–2994. 10.2174/0929867328666210915105929 34525905

[B23] Glauben R. Batra A. Stroh T. Erben U. Fedke I. Lehr H. A. (2008). Histone deacetylases: Novel targets for prevention of colitis-associated cancer in mice. Gut 57 (5), 613–622. 10.1136/gut.2007.134650 18194985

[B24] He Z. Yang D. Fan X. Zhang M. Li Y. Gu X. (2020). The roles and mechanisms of lncRNAs in liver fibrosis. Int. J. Mol. Sci. 21 (4), 1482. 10.3390/ijms21041482 32098245 PMC7073061

[B25] Hou J. He M. Chen Q. Liang S. (2022). LncRNA H19 acts as miR-301a-3p sponge to alleviate lung injury in mice with sepsis by regulating Adcy1. Immunopharmacol. Immunotoxicol. 44 (4), 565–573. 10.1080/08923973.2022.2067045 35438054

[B26] Korholz K. Ridinger J. Krunic D. Najafi S. Gerloff X. F. Frese K. (2021). Broad-spectrum HDAC inhibitors promote autophagy through FOXO transcription factors in neuroblastoma. Cells 10 (5), 1001. 10.3390/cells10051001 33923163 PMC8144997

[B27] Kuang Y. Bing Z. Jin X. Li Q. (2021). LncRNA H19 upregulation participates in the response of glioma cells to radiation. Biomed. Res. Int. 2021, 1728352. 10.1155/2021/1728352 34159190 PMC8187074

[B28] Lee H. Y. Tang D. W. Liu C. Y. Cho E. C. (2022). A novel HDAC1/2 inhibitor suppresses colorectal cancer through apoptosis induction and cell cycle regulation. Chem. Biol. Interact. 352, 109778. 10.1016/j.cbi.2021.109778 34929181

[B29] Li T. Zhang X. Cheng L. Li C. Wu Z. Luo Y. (2022). Modulation of lncRNA H19 enhances resveratrol-inhibited cancer cell proliferation and migration by regulating endoplasmic reticulum stress. J. Cell. Mol. Med. 26 (8), 2205–2217. 10.1111/jcmm.17242 35166018 PMC8995452

[B30] Li Y. Ma H. Y. Hu X. W. Qu Y. Y. Wen X. Zhang Y. (2020). LncRNA H19 promotes triple-negative breast cancer cells invasion and metastasis through the p53/TNFAIP8 pathway. Cancer Cell. Int. 20, 200. 10.1186/s12935-020-01261-4 32514245 PMC7257135

[B31] Li Y. Zhao K. Yao C. Kahwash S. Tang Y. Zhang G. (2016). Givinostat, a type II histone deacetylase inhibitor, induces potent caspase-dependent apoptosis in human lymphoblastic leukemia. Genes. Cancer 7 (9-10), 292–300. 10.18632/genesandcancer.117 28050230 PMC5115170

[B32] Liu H. C. Zhu W. Y. Ren L. Y. (2022). LncRNA H19 inhibits proliferation and enhances apoptosis of nephroblastoma cells by regulating the miR-675/TGFBI axis. Eur. Rev. Med. Pharmacol. Sci. 26 (11), 3800–3806. 10.26355/eurrev_202206_28947 35731049

[B33] Lo Dico A. Costa V. Martelli C. Diceglie C. Rajata F. Rizzo A. (2016). MiR675-5p acts on HIF-1α to sustain hypoxic responses: A new therapeutic strategy for glioma. Theranostics 6 (8), 1105–1118. 10.7150/thno.14700 27279905 PMC4893639

[B34] Mahmoudian-Sani M. R. Jalali A. Jamshidi M. Moridi H. Alghasi A. Shojaeian A. (2019). Long non-coding RNAs in thyroid cancer: Implications for pathogenesis, diagnosis, and therapy. Oncol. Res. Treat. 42 (3), 136–142. 10.1159/000495151 30799425

[B35] Minegaki T. Suzuki A. Mori M. Tsuji S. Yamamoto S. Watanabe A. (2018). Histone deacetylase inhibitors sensitize 5-fluorouracil-resistant MDA-MB-468 breast cancer cells to 5-fluorouracil. Oncol. Lett. 16 (5), 6202–6208. 10.3892/ol.2018.9388 30333885 PMC6176421

[B36] Mrakovcic M. Frohlich L. F. (2019). Molecular determinants of cancer therapy resistance to HDAC inhibitor-induced autophagy. Cancers (Basel) 12 (1), 109. 10.3390/cancers12010109 31906235 PMC7016854

[B37] Muller V. Oliveira-Ferrer L. Steinbach B. Pantel K. Schwarzenbach H. (2019). Interplay of lncRNA H19/miR-675 and lncRNA NEAT1/miR-204 in breast cancer. Mol. Oncol. 13 (5), 1137–1149. 10.1002/1878-0261.12472 30803129 PMC6487715

[B38] Nakagawa-Saito Y. Mitobe Y. Togashi K. Suzuki S. Sugai A. Kitanaka C. (2023). Givinostat inhibition of sp1-dependent MGMT expression sensitizes glioma stem cells to temozolomide. Anticancer Res. 43 (3), 1131–1138. 10.21873/anticanres.16258 36854532

[B39] O'Brien S. J. Scheurlen K. Rochet A. Fiechter C. Paas M. Pan J. (2022). Increased expression of long non-coding RNA H19 is associated with colon cancer recurrence. J. Surg. Res. 269, 59–68. 10.1016/j.jss.2021.08.004 34520983

[B40] Patra S. Panigrahi D. P. Praharaj P. P. Bhol C. S. Mahapatra K. K. Mishra S. R. (2019). Dysregulation of histone deacetylases in carcinogenesis and tumor progression: A possible link to apoptosis and autophagy. Cell. Mol. Life Sci. 76 (17), 3263–3282. 10.1007/s00018-019-03098-1 30982077 PMC11105585

[B41] Perego P. Zuco V. Gatti L. Zunino F. (2012). Sensitization of tumor cells by targeting histone deacetylases. Biochem. Pharmacol. 83 (8), 987–994. 10.1016/j.bcp.2011.11.010 22120677

[B42] Pramanik S. D. Kumar Halder A. Mukherjee U. Kumar D. Dey Y. N. R M. (2022). Potential of histone deacetylase inhibitors in the control and regulation of prostate, breast and ovarian cancer. Front. Chem. 10, 948217. 10.3389/fchem.2022.948217 36034650 PMC9411967

[B43] Psilopatis I. Pergaris A. Giaginis C. Theocharis S. (2021). Histone deacetylase inhibitors: A promising therapeutic alternative for endometrial carcinoma. Dis. Markers 2021, 7850688. 10.1155/2021/7850688 34804263 PMC8604582

[B44] Ramaiah M. J. Tangutur A. D. Manyam R. R. (2021). Epigenetic modulation and understanding of HDAC inhibitors in cancer therapy. Life Sci. 277, 119504. 10.1016/j.lfs.2021.119504 33872660

[B45] Rincon-Riveros A. Morales D. Rodriguez J. A. Villegas V. E. Lopez-Kleine L. (2021). Bioinformatic tools for the analysis and prediction of ncRNA interactions. Int. J. Mol. Sci. 22 (21), 11397. 10.3390/ijms222111397 34768830 PMC8583695

[B46] Roca M. S. Moccia T. Iannelli F. Testa C. Vitagliano C. Minopoli M. (2022). HDAC class I inhibitor domatinostat sensitizes pancreatic cancer to chemotherapy by targeting cancer stem cell compartment via FOXM1 modulation. J. Exp. Clin. Cancer Res. 41 (1), 83. 10.1186/s13046-022-02295-4 35241126 PMC8892808

[B47] Rodrigues Moita A. J. Bandolik J. J. Hansen F. K. Kurz T. Hamacher A. Kassack M. U. (2020). Priming with HDAC inhibitors sensitizes ovarian cancer cells to treatment with cisplatin and HSP90 inhibitors. Int. J. Mol. Sci. 21 (21), 8300. 10.3390/ijms21218300 33167494 PMC7663919

[B48] Saieva L. Barreca M. M. Zichittella C. Prado M. G. Tripodi M. Alessandro R. (2020). Hypoxia-induced miR-675-5p supports beta-catenin nuclear localization by regulating GSK3-beta activity in colorectal cancer cell lines. Int. J. Mol. Sci. 21 (11), 3832. 10.3390/ijms21113832 32481626 PMC7312749

[B49] Sandona M. Cavioli G. Renzini A. Cedola A. Gigli G. Coletti D. (2023). Histone deacetylases: Molecular mechanisms and therapeutic implications for muscular dystrophies. Int. J. Mol. Sci. 24 (5), 4306. 10.3390/ijms24054306 36901738 PMC10002075

[B50] Shermane Lim Y. W. Xiang X. Garg M. Le M. T. Li-Ann Wong A. Wang L. (2021). The double-edged sword of H19 lncRNA: Insights into cancer therapy. Cancer Lett. 500, 253–262. 10.1016/j.canlet.2020.11.006 33221454

[B51] Shima H. Kida K. Adachi S. Yamada A. Sugae S. Narui K. (2018). Lnc RNA H19 is associated with poor prognosis in breast cancer patients and promotes cancer stemness. Breast Cancer Res. Treat. 170 (3), 507–516. 10.1007/s10549-018-4793-z 29693231

[B52] Singh A. K. Bishayee A. Pandey A. K. (2018). Targeting histone deacetylases with natural and synthetic agents: An emerging anticancer strategy. Nutrients 10 (6), 731. 10.3390/nu10060731 29882797 PMC6024317

[B53] Spreafico M. Cafora M. Bragato C. Capitanio D. Marasca F. Bodega B. (2021). Targeting HDAC8 to ameliorate skeletal muscle differentiation in Duchenne muscular dystrophy. Pharmacol. Res. 170, 105750. 10.1016/j.phrs.2021.105750 34214631

[B54] Squarzoni A. Scuteri A. Cavaletti G. (2022). HDACi: The columbus' egg in improving cancer treatment and reducing neurotoxicity? Cancers (Basel) 14 (21), 5251. 10.3390/cancers14215251 36358670 PMC9654569

[B55] Suraweera A. O'Byrne K. J. Richard D. J. (2018). Combination therapy with histone deacetylase inhibitors (HDACi) for the treatment of cancer: Achieving the full therapeutic potential of HDACi. Front. Oncol. 8, 92. 10.3389/fonc.2018.00092 29651407 PMC5884928

[B56] Taiarol L. Bigogno C. Sesana S. Kravicz M. Viale F. Pozzi E. (2022). Givinostat-Liposomes: Anti-Tumor effect on 2D and 3D glioblastoma models and pharmacokinetics. Cancers (Basel) 14 (12), 2978. 10.3390/cancers14122978 35740641 PMC9220922

[B57] Tang Z. Liu L. Borlak J. (2023). Combined inhibition of histone deacetylase and cytidine deaminase improves epigenetic potency of decitabine in colorectal adenocarcinomas. Clin. Epigenetics 15 (1), 89. 10.1186/s13148-023-01500-1 37208732 PMC10199547

[B58] Vagapova E. Kozlov M. Lebedev T. Ivanenko K. Leonova O. Popenko V. (2021). Selective inhibition of HDAC class I sensitizes leukemia and neuroblastoma cells to anticancer drugs. Biomedicines 9 (12), 1846. 10.3390/biomedicines9121846 34944663 PMC8698907

[B59] Vojinovic J. Damjanov N. D'Urzo C. Furlan A. Susic G. Pasic S. (2011). Safety and efficacy of an oral histone deacetylase inhibitor in systemic-onset juvenile idiopathic arthritis. Arthritis Rheum. 63 (5), 1452–1458. 10.1002/art.30238 21538322

[B60] Vojinovic J. Damjanov N. (2011). HDAC inhibition in rheumatoid arthritis and juvenile idiopathic arthritis. Mol. Med. 17 (5-6), 397–403. 10.2119/molmed.2011.00030 21308151 PMC3105145

[B61] Wang M. Han D. Yuan Z. Hu H. Zhao Z. Yang R. (2018). Long non-coding RNA H19 confers 5-Fu resistance in colorectal cancer by promoting SIRT1-mediated autophagy. Cell. Death Dis. 9 (12), 1149. 10.1038/s41419-018-1187-4 30451820 PMC6242979

[B62] Wang Y. Wu Z. Li Y. Zheng Z. Yan J. Tian S. (2020). Long non-coding RNA H19 promotes proliferation, migration and invasion and inhibits apoptosis of breast cancer cells by targeting miR-491-5p/znf703 Axis. Cancer Manag. Res. 12, 9247–9258. 10.2147/CMAR.S246009 33061615 PMC7532042

[B63] Xiao Q. Liu H. Wang H. S. Cao M. T. Meng X. J. Xiang Y. L. (2020). Histone deacetylase inhibitors promote epithelial-mesenchymal transition in Hepatocellular Carcinoma via AMPK-FOXO1-ULK1 signaling axis-mediated autophagy. Theranostics 10 (22), 10245–10261. 10.7150/thno.47045 32929346 PMC7481427

[B64] Xu J. Xia Y. Zhang H. Guo H. Feng K. Zhang C. (2018). Overexpression of long non-coding RNA H19 promotes invasion and autophagy via the PI3K/AKT/mTOR pathways in trophoblast cells. Biomed. Pharmacother. 101, 691–697. 10.1016/j.biopha.2018.02.134 29522949

[B65] Yang F. Bi J. Xue X. Zheng L. Zhi K. Hua J. (2012). Up-regulated long non-coding RNA H19 contributes to proliferation of gastric cancer cells. FEBS J. 279 (17), 3159–3165. 10.1111/j.1742-4658.2012.08694.x 22776265

[B66] Yang H. Lin H. C. Liu H. Gan D. Jin W. Cui C. (2020). A 6 lncRNA-based risk score System for predicting the recurrence of colon adenocarcinoma patients. Front. Oncol. 10, 81. 10.3389/fonc.2020.00081 32117736 PMC7015976

[B67] Yang H. (2023). Silencing of long non-coding RNA H19 alleviates lipopolysaccharide (LPS)-induced apoptosis and inflammation injury by regulating miR-140-5p/TLR4 Axis in cell models of pneumonia. Curr. Mol. Med. 23 (3), 275–284. 10.2174/1566524022666220407100949 35392782

[B68] Yang J. Qi M. Fei X. Wang X. Wang K. (2021). LncRNA H19: A novel oncogene in multiple cancers. Int. J. Biol. Sci. 17 (12), 3188–3208. 10.7150/ijbs.62573 34421359 PMC8375239

[B69] Yang W. Ning N. Jin X. (2017). The lncRNA H19 promotes cell proliferation by competitively binding to miR-200a and derepressing beta-catenin expression in colorectal cancer. Biomed. Res. Int. 2017, 2767484. 10.1155/2017/2767484 28164117 PMC5259610

[B70] Yang W. Redpath R. E. Zhang C. Ning N. (2018). Long non-coding RNA H19 promotes the migration and invasion of colon cancer cells via MAPK signaling pathway. Oncol. Lett. 16 (3), 3365–3372. 10.3892/ol.2018.9052 30127936 PMC6096146

[B71] Ye Y. Shen A. Liu A. (2019). Long non-coding RNA H19 and cancer: A competing endogenous RNA. Bull. Cancer 106 (12), 1152–1159. 10.1016/j.bulcan.2019.08.011 31753509

[B72] Yokoyama Y. Sakatani T. Wada R. Ishino K. Kudo M. Koizumi M. (2019). *In vitro* and *in vivo* studies on the association of long non-coding RNAs H19 and urothelial cancer associated 1 with the susceptibility to 5-fluorouracil in rectal cancer. Int. J. Oncol. 55 (6), 1361–1371. 10.3892/ijo.2019.4895 31638183

[B73] Yuan W. Huang J. Hou S. Li H. Bie L. Chen B. (2022). The antigastric cancer effect of Triptolide is associated with H19/NF-κB/FLIP Axis. Front. Pharmacol. 13, 918588. 10.3389/fphar.2022.918588 36110523 PMC9469193

[B74] Zhang B. Lin J. Zhang J. Wang X. Deng X. (2022a). Integrated chromatin accessibility and transcriptome landscapes of 5-fluorouracil-resistant colon cancer cells. Front. Cell. Dev. Biol. 10, 838332. 10.3389/fcell.2022.838332 35252200 PMC8891516

[B75] Zhang R. Xu J. Zhao J. Liu F. (2017). Upregulated serum miR-675 predicts poor prognosis for colorectal cancer. Int. J. Clin. Exp. Pathol. 10 (7), 8043–8049. 31966656 PMC6965284

[B76] Zhang X. Luo M. Zhang J. Guo B. Singh S. Lin X. (2022b). The role of lncRNA H19 in tumorigenesis and drug resistance of human Cancers. Front. Genet. 13, 1005522. 10.3389/fgene.2022.1005522 36246634 PMC9555214

[B77] Zhao C. Dong H. Xu Q. Zhang Y. (2020). Histone deacetylase (HDAC) inhibitors in cancer: A patent review (2017-present). Expert Opin. Ther. Pat. 30 (4), 263–274. 10.1080/13543776.2020.1725470 32008402

[B78] Zhao W. Lin X. Han H. Zhang H. Li X. Jiang C. (2021a). Long noncoding RNA H19 contributes to the proliferation and autophagy of glioma cells through mTOR/ULK1 pathway. Neuroreport 32 (5), 352–358. 10.1097/WNR.0000000000001602 33661803

[B79] Zhao X. Jin X. Zhang Q. Liu R. Luo H. Yang Z. (2021b). Silencing of the lncRNA H19 enhances sensitivity to X-ray and carbon-ions through the miR-130a-3p/WNK3 signaling axis in NSCLC cells. Cancer Cell. Int. 21 (1), 644. 10.1186/s12935-021-02268-1 34863180 PMC8642868

[B80] Zhou H. Wang B. Yang Y. X. Jia Q. J. Zhang A. Qi Z. W. (2019). Long noncoding RNAs in pathological cardiac remodeling: A review of the update literature. Biomed. Res. Int. 2019, 7159592. 10.1155/2019/7159592 31355277 PMC6634064

[B81] Zhuang Y. Li T. Xiao H. Wu J. Su S. Dong X. (2021). LncRNA-H19 drives cardiomyocyte senescence by targeting miR-19a/socs1/p53 Axis. Front. Pharmacol. 12, 631835. 10.3389/fphar.2021.631835 33664669 PMC7921730

[B82] Zichittella C. Barreca M. M. Cordaro A. Corrado C. Alessandro R. Conigliaro A. (2022). Mir-675-5p supports hypoxia-induced drug resistance in colorectal cancer cells. BMC Cancer 22 (1), 567. 10.1186/s12885-022-09666-2 35596172 PMC9123752

