# Supplementary material for: Long non-coding RNA H19 enhances the pro-apoptotic activity of ITF2357 (a histone deacetylase inhibitor) in colorectal cancer cells
Source: Front Pharmacol. 2023 Sep 28;14:1275833. doi: 10.3389/fphar.2023.1275833 (PMC10572549; doi:10.3389/fphar.2023.1275833)
Supplement: Supplementary file 1 [file Table1.DOCX]

Supplementary Material

Long non-coding RNA H19 enhances the pro-apoptotic activity of ITF2357 (Histone Deacetylase Inhibitor) in colorectal cancer cells

Chiara Zichittella^1^, Marco Loria^1^, Adriana Celesia^2^, Diana Di Liberto^2^, Chiara Corrado^1^, Riccardo Alessandro^1, 3^, Sonia Emanuele^2*^, Alice Conigliaro^1*^.

^1^ Department of Biomedicine, Neurosciences and Advanced Diagnostics (Bi.N.D.), Section of Biology and Genetics, University of Palermo, 90133 Palermo, Italy.

^2^ Department of Biomedicine, Neurosciences and Advanced Diagnostics (Bi.N.D.), Biochemistry Building, University of Palermo, 90127 Palermo, Italy.

^3^ Institute for Biomedical Research and Innovation (IRIB), National Research Council (CNR), 90146 Palermo, Italy.

*** Correspondence:**Corresponding Authors: Alice Conigliaro and Sonia Emanuele

[alice.conigliaro@unipa.it](mailto:alice.conigliaro@unipa.it)

[sonia.emanuele@unipa.it](mailto:sonia.emanuele@unipa.it)

**
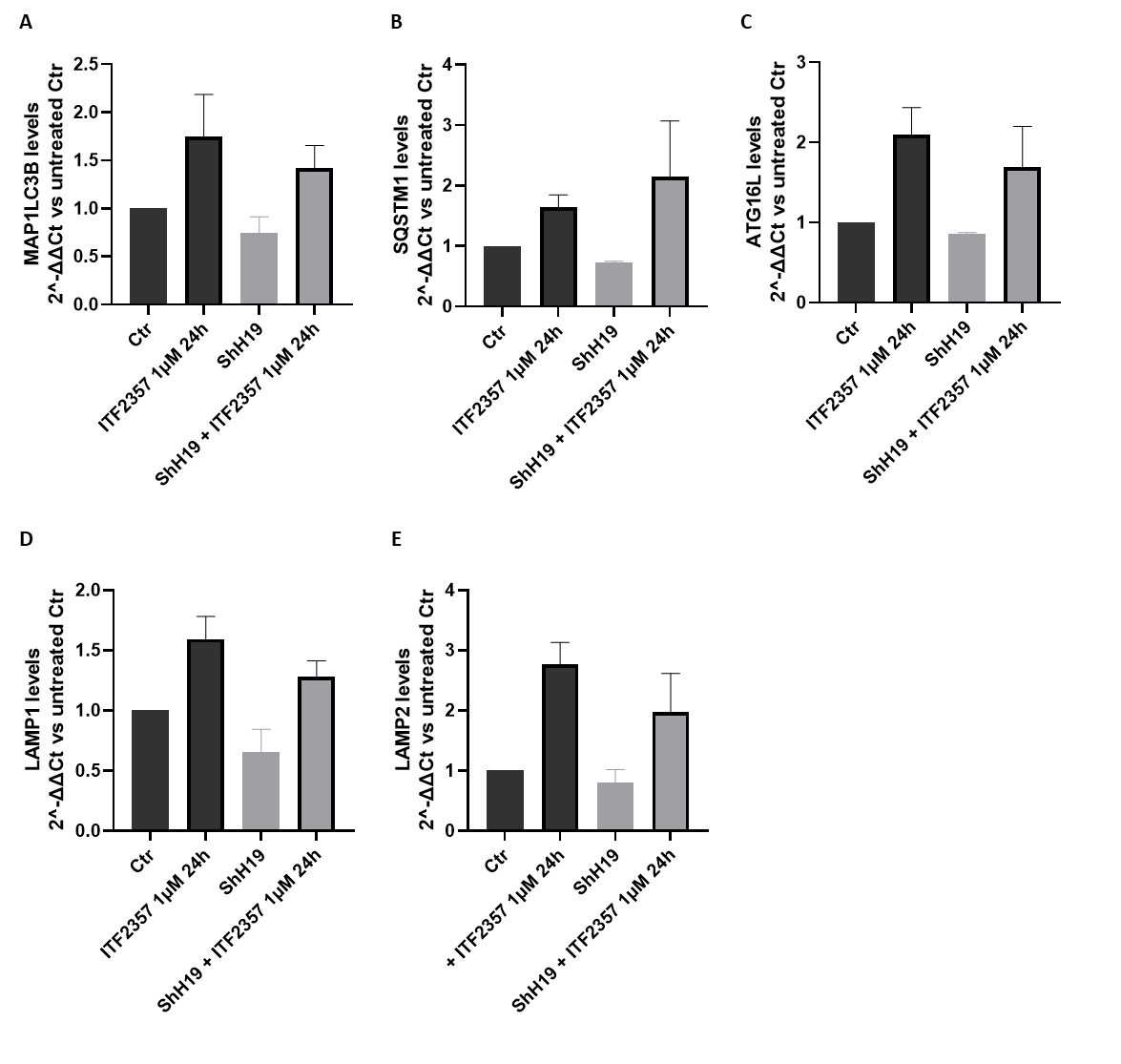
**

***Supplementary Figure 1: LncH19 silencing did not affect ITF2357-induced autophagy.***

**A**-**E**: Analysis of the expression level (qRT-PCR) of autophagic genes in HCT-116 silenced for lncH19 or control cells (Ctr) untreated or treated with 1 µM concentration of ITF2357 for 24 hours. The expression levels of genes are reported as 2^-ΔΔCt compared to control cells (Ctr), threshold cycle were normalized against β-actin. Data are expressed as the mean ± SD.
