# Supplementary figures and images for: Long non-coding RNA H19 enhances the pro-apoptotic activity of ITF2357 (a histone deacetylase inhibitor) in colorectal cancer cells
Source: Front Pharmacol. 2023 Sep 28;14:1275833. doi: 10.3389/fphar.2023.1275833 (PMC10572549; doi:10.3389/fphar.2023.1275833)

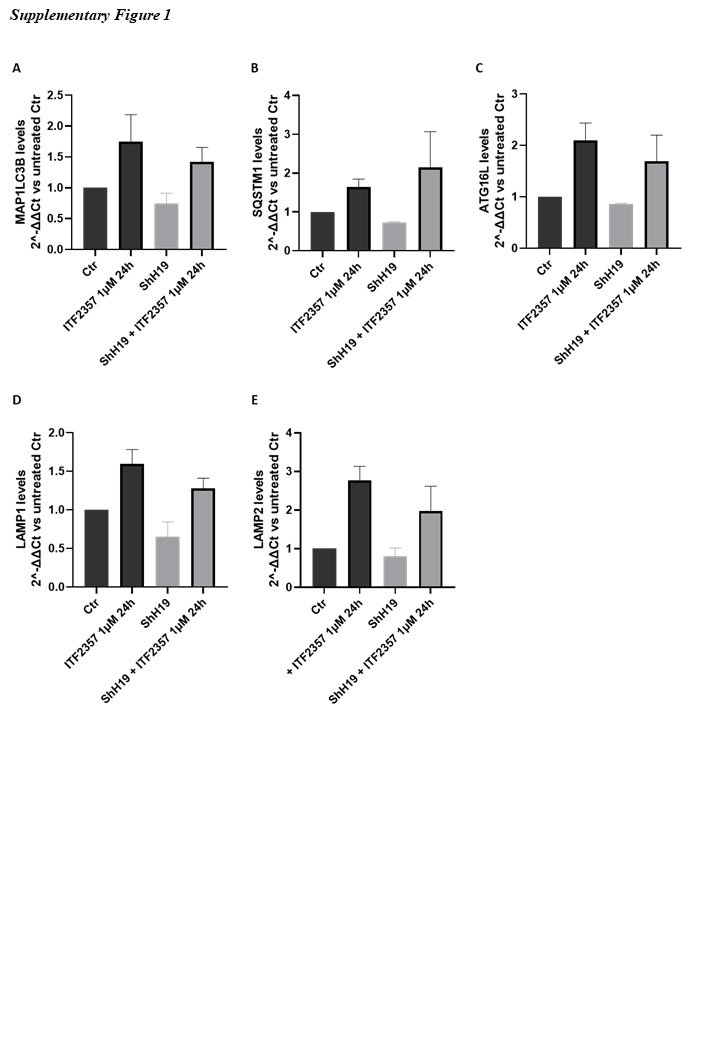

Supplement: Supplementary file 2 [file Image1.TIF]
